# *Cannabis sativa* and/or melatonin do not alter brain lipid but alter oxidative mechanisms in female rats

**DOI:** 10.1186/s42238-021-00095-9

**Published:** 2021-08-19

**Authors:** Halimat Amin Abdulrahim, Isiaka Abdullateef Alagbonsi, Oluwasola Amuda, Noah Adavize Omeiza, Abdul-Rahuf Aderemi Feyitimi, Luqman Aribidesi Olayaki

**Affiliations:** 1 Department of Medical Biochemistry, College of Health Sciences, University of Ilorin, Ilorin, Nigeria; 2 Department of Clinical Biology (Physiology unit), School of Medicine and Pharmacy, College of Medicine and Health Sciences, University of Rwanda, Huye, Rwanda; 3 Department of Medical Laboratory Sciences, Faculty of Health Sciences, Al-Hikmah University, Ilorin, Kwara P.M.B. 1601 Nigeria; 4 Department of Physiology, College of Health Sciences, University of Ilorin, Ilorin, Nigeria

**Keywords:** Brain, *Cannabis sativa*, Lipid, Melatonin, Oxidative stress

## Abstract

**Background:**

Lipid profile and redox status play a role in brain (dys)functions. Cannabinoid and melatonergic systems operate in the brain and contribute to brain (patho)physiology, but their roles in the modulation of brain lipid and redox status are not well-known. We studied the effect of ethanol extract of *Cannabis sativa* (CS) and/or melatonin (M) on the lipid profile and anti-oxidant system of the rat brain.

**Methods:**

We randomly divided twenty-four (24) female Wistar rats into 4 groups (*n* = 6 rats each). Group 1 (control) received distilled water mixed with DMSO. Groups II–IV received CS (2 mg/kg), M (4 mg/kg), and co-administration of CS and M (CS + M) respectively via oral gavage between 8:00 am and 10:00 am once daily for 14 days. Animals underwent 12-h fasting after the last day of treatment and sacrificed under ketamine anesthesia (20 mg/kg; i.m). The brain tissues were excised and homogenized for assay of the concentrations of the total cholesterol (TC), triacylglycerol (TG), high-density lipoprotein cholesterol (HDL-C), nitric oxide (NO), malondialdehyde (MDA), and the activities of glucose-6-phosphate dehydrogenase (G6PD), glutathione reductase (GR), glutathione peroxidase (GPx), catalase (CAT), superoxide dismutase (SOD), and acetylcholinesterase (AChE). One-way analysis of variance (ANOVA) was used to compare means across groups, followed by the least significant difference (LSD) post-hoc test.

**Results:**

CS and/or M did not affect the lipid profile parameters. However, CS increased the G6PD (from 15.58 ± 1.09 to 21.02 ± 1.45 U/L; *p* = 0.047), GPx (from 10.47 ± 0.86 to 17.71 ± 1.04 U/L; *p* = 0.019), and SOD (from 0.81 ± 0.02 to 0.90 ± 0.01 μM; *p* = 0.007), but decreased NO (from 9.40 ± 0.51 to 6.75 ± 0.21 μM; *p* = 0.010) and had no effect on MDA (*p* = 0.905), CAT (*p* = 0.831), GR (*p* = 0.639), and AChE (*p* = 0.571) in comparison with the control group. M augmented the increase in G6PD (from 21.02 ± 1.45 U/L to 27.18 ± 1.81 U/L; *p* = 0.032) and decrease in NO (from 6.75 ± 0.21 to 4.86 ± 0.13 μM; *p* = 0.034) but abolished the increase in GPx (from 17.71 ± 1.04 to 8.59 ± 2.06 U/L; *p* = 0.006) and SOD (from 0.90 ± 0.01 to 0.70 ± 0.00 μM; *p* = 0.000) elicited by CS in the rat brain in comparison with the CS group.

**Conclusions:**

CS and M do not alter brain lipid profile. Our data support the contention that CS elicits an anti-oxidative effect on the brain tissue and that CS + M elicits a pro-oxidant effect in rat brain.

**Supplementary Information:**

The online version contains supplementary material available at 10.1186/s42238-021-00095-9.

## Introduction

The brain tissue is vulnerable to oxidative damage due to its richness in polyunsaturated fatty acids (PUFAs), presence of redox-active metals like Fe2+ and Cu2+, and high oxygen consumption rate. Unfortunately, the brain has limited antioxidant mechanisms compared to other organs (Floyd, 1999). Thus, brain oxidative stress contributes to the pathogenesis of various neurodegenerative disorders. In fact, epileptogenic agents, including Pilocarpine and pentylenetetrazole, have been shown to cause elevated reactive oxygen species (ROS) in the brain of rats (Freitas, 2009). Neural tissues or sera from amyotrophic lateral sclerosis (ALS) patients and transgenic mice expressing mutant SOD1 have been reported to exhibit increased deoxyribonucleic acid (DNA) damage and lipid peroxidation (Simpson et al., 2004).

The effects of cannabinoids (CBs) on the lipid metabolism and redox system in the brain have been reported but not well understood. For instance, tetrahydrocannabinol (THC)-enriched *Cannabis sativa* (CS) extract exacerbates epileptic seizures and increased ROS and lipids induced by pentylenetetrazole in rat brain (Abdel-Salam et al., 2018). Melatonin (*N*-acetyl-5-methoxytryptamine, M) is a ubiquitous bioactive molecule that is secreted by the pineal and other organs in mammals (Acuña-Castroviejo et al., 2014) and is present in bacteria, fungi, animals, and plants (Cipolla-Neto et al., 2014). Aside from maintaining circadian rhythms, M also performs antioxidant (Reiter et al., 2018), anti-apoptotic (Amin et al., 2015), anti-inflammatory (Hardeland, 2018), analgesic (Yang et al., 2018), and anti-cancer (Reiter et al., 2017) functions. Interestingly, M also has a neuroprotective effect in cases of ischemic stroke and traumatic brain injury (Zhao et al., 2018) and beneficial effects on several central nervous system (CNS) disorders including Alzheimer’s disease (Corpas et al., 2018) and cognitive impairments (Song et al., 2014). Several mechanisms for the neuroprotective effect of M have been proposed, some of which include antioxidant (Tan et al., 2015), anti-apoptosis (El-Missiry et al., 2014), improvement of mitochondrial functioning (Tan et al., 2016), and antagonism of brain insulin resistance (Xu et al., 2019).

The crosstalk between CB-melatonergic systems has been well-established. In the testis, for instance, we have extensively shown that M exacerbates CB-induced gonadotoxicity in-vivo but ameliorates it in-vitro (Alagbonsi & Olayaki, 2018; Alagbonsi & Olayaki, 2017; Alagbonsi et al., 2016; Alagbonsi & Olayaki, 2016). Cannabinoid receptors (CBRs), especially the subtype 1, are predominantly expressed in the CNS (Svizenska et al., 2008) where they regulate synaptic functions, memory, and motor learning (Pacher et al., 2006). The CBs, including THC, cannabidiol, and cannabinol, have been shown to attenuate the norepinephrine-induced stimulation of M biosynthesis in the rat pineal gland by reducing arylalkylamine *N*-acetyltransferase (AANAT) activity, even though this effect is not mediated by CBRs 1 and 2 (Koch et al., 2006). It was further shown that the CB-dependent attenuation of M biosynthesis and AANAT activity is mediated by an intracellular interaction between CBs and the activated AANAT enzyme (Koch et al., 2006).

Development of epileptic seizures and brain tissue lipid peroxidation are ameliorated by antioxidants like vitamin C (Ayyildiz et al., 2007), resveratrol (Mishra et al., 2015), β-caryophyllene (de Oliveira et al., 2016), and quercetin (Sefil et al., 2014). Cognition impairment in high-fat diet (HFD)-fed aged rats was alleviated by through antagonism of brain insulin resistance (Xu et al., 2019). The CBs and M are also endogenously produced in mammals and other animals. Since lipid profile and redox status have been reported to modulate brain physiology and pathophysiology, and the CBs and melatonergic systems are predominant in the brain, there is a possibility that these systems could have synergistic, additive, or even antagonistic effects on the brain lipid and anti-oxidant profile. Presently, there is no clear information about the effect of co-administration of CS and M on brain lipid and redox system.

Dim light M onset, a highly utilized marker of circadian rhythm that represents the time at which M level begins to rise, has been reported to occur significantly earlier in women than in men (Van Reen et al., 2013). Using constant routine protocols in humans, females have also been reported to exhibit greater level of plasma M than males (Gunn et al., 2016). In a study that investigated the regulation of cognition by circadian rhythm and sleep-wake cycle, it was reported that the amplitude of circadian modulation is higher in women than in men (Santhi et al., 2016). In addition, studies have reported that women show higher CBR1 protein expression (Onaivi et al., 1999) and are more susceptible to CB-induced visuospatial memory impairment and hemodynamic changes than men (Mathew et al., 2003). Female rats also demonstrate higher acquisition, maintenance, response, locomotor, anti-nociceptive, and cataleptic effects than male rats after administration of CBs (Tseng & Craft, 2004) while administration of CBs are also more acquired, maintained. These established gender difference in the effect of CS and M informed our choice of female rats.

In this study, we studied the effect of CS and/or M on the lipid profile and anti-oxidant system in the female rats’ brain. We hypothesized that CS will alter the brain lipid and redox status, which will be ameliorated by M.

## Materials and methods

### Animal care

We obtained twenty-four (24) adult female Wistar rats (150–170 g) from the Department of Biochemistry, University of Ilorin, Nigeria and housed them in plastic cages under the condition of uniform humidity (65%) and temperature (22–25 °C) on a 12-h light-dark cycle. They were fed with a standard rodent pelletized diet (Ace Feeds, Ibadan, Nigeria) with free access to distilled water ad libitum, and were exposed to a 7-day acclimatization period before the commencement of the treatments. In addition to the care of the animals according to the National Academy of Science guidelines, USA (Albus, 2012), the study protocol was approved by the University of Ilorin Ethical Research Committee with clearance code: UERC/ASN/2018/1152.

### Extraction of CS leaves

The dried leaves of CS, sourced from Uzeba village in Edo State, Nigeria, were kindly donated by the National Drug Law Enforcement Agency (NDLEA), Ilorin office, Kwara State, Nigeria for research purpose only. The leaves were ground into a powder with a manual mortar and pestle (700 g obtained). The pulverized product was divided into two parts; one part was used for extraction and chromatographic analysis while the other part was used for proximate analysis and phytochemical studies respectively. Extraction of CS was done with Soxhlet apparatus by soaking 300 g in 98% ethanol for 8 hours as previously described (Mandal & Das, 2010). It was filtered and a rotary evaporator was then used to evaporate the filtrate to dryness (dried filtrate weighed 45.2 g and its percentage yield was 15.1%).

### Experimental design

Determination of the lethal dose that kills 50% of the treated animals (LD50) was done as previously described (Yassa et al., 2010) and one-tenth (1/10th) of the LD50 was calculated to be 2 mg/kg, which was considered as the experimental dose. Based on an established animal sampling method (Charan & Kantharia, 2013), the rats were randomly divided in a blinded fashion into 4 groups (*n* = 6 rats each). Stock solutions of the CS and M were prepared with dimethyl sulphoxide (DMSO), which served as the vehicle for their oral administration. The solutions were stored at 20 °C and diluted to the required concentration to achieve the needed dose for each animal. Group 1 received control mixture (distilled water mixed with DMSO, making 0.2% DMSO, vol/vol) and served as the control. Groups II–IV received an ethanol extract of *Cannabis sativa* (CS, 2 mg/kg); 4 mg/kg M (Bulk Supplements, Henderson, Nevada, USA); and co-administration of CS and M (CS + M) via oral gavage between 8:00 am and 10:00 am once daily for 14 days. In our preliminary experiment to confirm the effect of control mixture, we assayed the parameters reported herein in the brain of rats that did not receive the control mixture but only distilled water in addition to standard diet for the same duration of 14 days. We compared the data of these animals with those of the control that received the control mixture using the *T* test and found that the control mixture had no statistical significance on the parameters assessed in this study.

Animals underwent 12-h fasting after the last day of treatment and sacrificed under ketamine anesthesia (20 mg/kg; i.m). The brain tissues were excised, rinsed with normal saline, and homogenized with a mechanized homogenizer in 30 % chilled sucrose (1:4 ratio w/v); centrifuged at 4000 rpm for 15 min to sediment nuclei and cell debris, and the resulting supernatants were collected in plain bottles and stored at − 20 °C before biochemical analyses.

### Biochemical parameters

Brain homogenates were used to determine the concentrations of the total cholesterol (TC), triglyceride (TG), high-density lipoprotein-cholesterol (HDL-C) (Richmond, 1976), nitric oxide (NO) (Guevara et al., 1998), malondialdehyde (MDA) (Ohkawa et al., 1979), and the activities of glucose-6-phosphate dehydrogenase (G6PD) (Lohr & Waller, 1974), glutathione reductase (GR) (Cribb et al., 1989), glutathione peroxidase (GPx) (Pleban et al., 1982), catalase (CAT) (Claiborne, 1985), SOD (Kakkar et al., 1984), and ACh (Ellman et al., 1961). TC, TG, and HDL-C levels were assessed using Labkit® diagnostic kit reagents as described by the manufacturer. Thereafter, very low-density lipoprotein-cholesterol (VLDL-C) was estimated as one-fifth of TG and low-density lipoproteins-cholesterol (LDL-C) was calculated using Friedward formula: [LDL-C = TC – (HDL-C + VLDL) mg/dl]. Additionally, the following lipid indices were estimated thus: cardiovascular risk ratio (CRR): CRRI = TC/HDL-C ratio, CRRII = LDL-C/HDL-C ratio; Non-HDL-C = TC-HDL-C (measures all lipoprotein containing cholesterol), atherogenic coefficient (AC) = non-HDL-C/HDL-C ratio (measures the risk of coronary artery disease); and atherogenic index of plasma (AIP) = log TG/HDL-C (Dobiášová, 2004). AIP is a novel index that has been used as an optimal indicator of dyslipidemia and associated conditions (Zhao et al., 2017).

Information about the assay methods, manufacturers, product codes, and products’ sensitivity and specificity are summarized in Table 1.

**Table 1:** Product information on biochemical assays

	Parameter	Methods	Manufacturers	Product code	Sensitivity
1	TC (mg/100 mg tissue)	Enzymatic colorimetric test	Fortress Diagnostics, Antrim, Northern Ireland, UK	BXC0261	0.20 mmol/l (7.74 mg/dl)
2	TG (mg/100 mg tissue)	Enzymatic colorimetric test	Fortress Diagnostics, Antrim, Northern Ireland, UK	BXC0271	0.05 mmol/l (3 mg/dl)
3	HDL (mg/100 mg tissue)	Enzymatic colorimetric test	Fortress Diagnostics, Antrim, Northern Ireland, UK	BXC0421	0.02 mmol/l (0.9 mg/dl)
4	G6PD (U/L)	UV/enzymatic	Spectrum Diagnostics, Egyptian Company for Biotechnology, Cairo, Egypt	REF:372002	19.5 U/g Hb
5	NO (μM)	Colorimetric	Elabscience Houston, Texas, USA	E-BC-K035-S	0.16 μmol/L
6	MDA (μM)	Colorimetric TBARS microplate Assay	Oxford Biomedical Research, Rochester Hills, MI, USA	FR40.130619	1 μm
7	GR (U/L)	Colorimetric	Abcam, Cambridge, UK	Ab83461	0.1 mU/ml
8	GPx (U/L)	UV/enzymatic	Fortress Diagnostics, Northern Ireland, UK	BXC0551	0.1 mU/ml
9	CAT (U/L)	Colorimetric	Mybiosource, Sunny Southern California, San Diego, USA.	MBS726781	0.1 ng/ml
10	SOD (μM)	Colorimetric	Mybiosource Sunny Southern California, San Diego, USA.	MBS2707324	0.067 ng/ml
11	AChE (μmolAcSCH/min/mg protein)	Colorimetric	Abcam, Cambridge, UK	Ab138871	1 mU/ml

### Statistical analysis

Data from all the animals in each group were used for statistical analysis and were represented as Mean ± SEM after analysis with version 20 of Statistical Package for Social Sciences (SPSS) (IBM Corporation, Armonk, NY). One-way analysis of variance (ANOVA) was used to compare means across groups (unless where otherwise stated), followed by the least significant difference (LSD) post-hoc test. *p* ≤ 0.05 was considered as statistically significant.

## Results

### Proximate and phytochemical screening of CS

Results of the proximate analysis, phytochemical screening, and chromatographic examination of CS are presented in the supplementary data (Alagbonsi et al. 2019).

### Effect of CS and/or M on lipid profiles and indices in brain

**Fig. 1:**
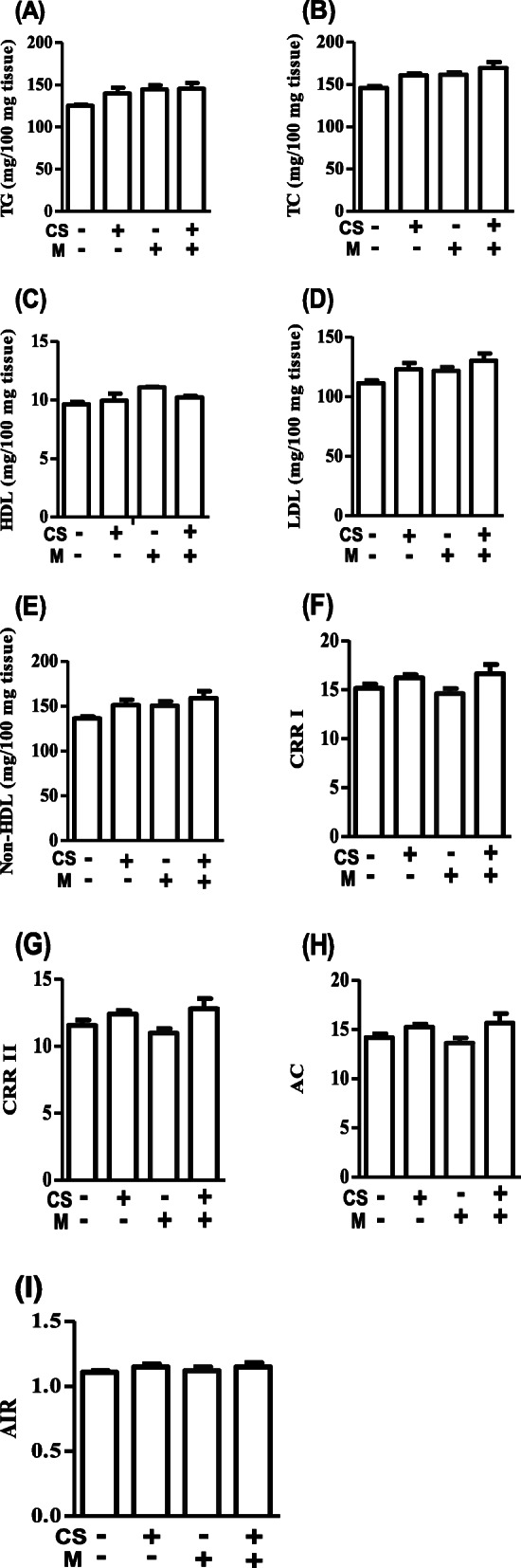
CS and M do not alter brain lipid either when administered separately or when co-administered in female rats. **A**–**I** represent TG, triglyceride; TC, total cholesterol; HDL, high-density lipoprotein; LDL, low-density lipoprotein; non-HDL, non- high density lipoprotein; CRRI, cardiovascular risk ratio I; CRRII, cardiovascular risk ratio II; AC, atherogenic coefficient; and AIR, atherogenic index ratio respectively. CS, ethanol extract of *Cannabis sativa*; M, melatonin; -, not administered; +, administered

The CS and/or M did not affect the TG (Fig. 1A), TC (Fig. 1B), HDL (Fig. 1C), LDL (Fig. 1D), non-HDL (Fig. 1E), CRRI (Fig. 1F), CRRII (Fig. 1G), AC (Fig. 1H), and AIR (Fig. 1I) of rats’ brains as their omnibus ANOVA results were insignificant (*p* > 0.05). These showed that CS and M do not have effect of rats’ brain lipid profile either when administered separately or when combined.

### Effect of CS and/or M on the pro-oxidant-antioxidant system in the brain

**Fig. 2:**
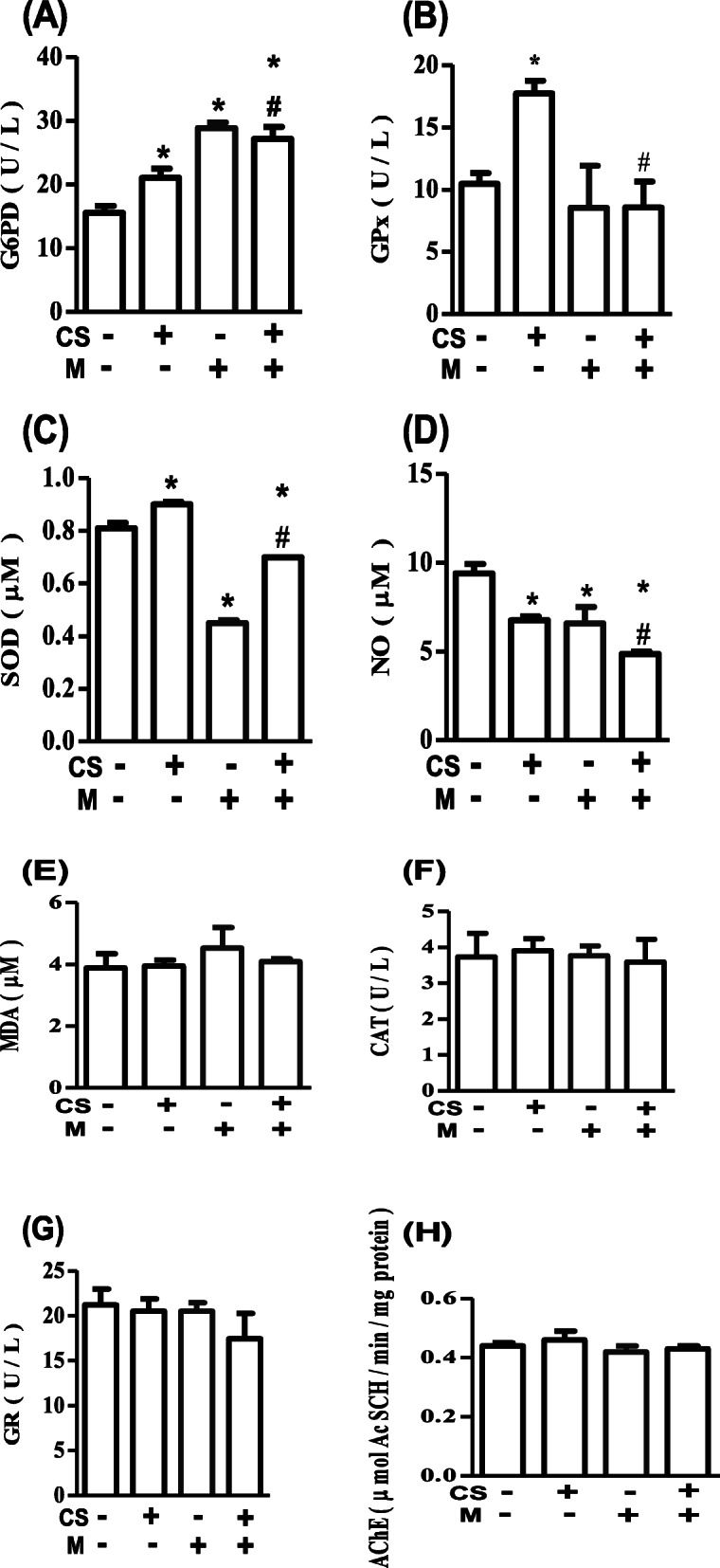
M augments CS-induced increase in G6PD and decrease in NO but abolishes the CS-induced increase in GPx and SOD. **A**–**H** represent G6PD, glucose-6-phosphate dehydrogenase; GPx, glutathione peroxidase; SOD, superoxide dismutase; NO, nitric oxide; MDA, malondialdehyde; CAT, catalase; GR, glutathione reductase; and AChE, acetylcholinesterase respectively. *p < 0.05 vs. control; ^#^*p* < 0.05 vs. CS; CS, ethanol extract of *Cannabis sativa*; M, melatonin; -, not administered; +, administered

The omnibus ANOVA result of the G6PD was significant (*p* = 0.007). Furthermore, the post-hoc test for multiple comparisons showed that the G6PD was significantly increased in the brain of rats that received CS (21.02 ± 1.45 U/L; *p* = 0.047), M (28.86 ± 0.86 U/L; *p* = 0.002), and CS + M (27.18 ± 1.81 U/L; *p* = 0.004) when compared to control group (15.58 ± 1.09 U/L). Moreover, CS + M caused more increase in the brain G6PD than CS only (*p* = 0.032) (Fig. 2A).

The omnibus ANOVA result of the GPx was significant (*p* = 0.021). Furthermore, the post-hoc test for multiple comparisons showed that the GPx was significantly increased by CS (17.71 ± 1.04 U/100 mg tissue; *p* = 0.019) but unaffected by M (8.55 ± 3.38 U/100 mg tissue; *p* = 0.494), while the CS-induced increase in GPx was abolished by M (8.59 ± 2.06 U/100 mg tissue; *p* = 0.455) when compared to control (10.47 ± 0.86 U/100 mg tissue) (Fig. 2B).

The omnibus ANOVA result of the SOD was significant (*p* = 0.000). Furthermore, the post-hoc test for multiple comparisons showed that the SOD was significantly increased by CS (0.90 ± 0.01 U/100 mg tissue; *p* = 0.007) but decreased by M (0.45 ± 0.01 U/100 mg tissue; *p* = 0.000) and CS + M (0.70 ± 0.00 U/100 mg tissue; *p* = 0.002) when compared to control (0.81 ± 0.02 U/100 mg tissue) (Fig. 2C).

The omnibus ANOVA result of the NO was significant (*p* = 0.007). Furthermore, the post-hoc test for multiple comparisons showed that the NO was significantly reduced in the brain of rats that received CS (6.75 ± 0.21 μM; *p* = 0.010), M (6.57 ± 0.93 μM; *p* = 0.011), and CS + M (4.86 ± 0.13 μM; *p* = 0.001) when compared to control (9.40 ± 0.51 μM). Moreover, CS + M caused more decrease in the brain NO than CS only (*p* = 0.034) (Fig. 2D).

The CS and/or M did not affect the MDA (Fig. 2E), CAT (Fig. 2F), GR (Fig. 2G), and AChE (Fig. 2H) of rats’ brains, as their omnibus ANOVA results were not significant (*p* > 0.05).

## Discussion

Since cholesterol is not transported from the plasma into the brain through the blood-brain barrier, most of the cholesterols in the brain (over 95%) are produced from de novo synthesis in the glia (astrocytes), neurons, and oligodendrocytes (Dietschy, 2009). The synthesized cholesterol will then be internalized in the endosome/lysosome system after its secretion via transport molecules and uptake by neurons’ lipoprotein receptors. Consequently, Niemann-Pick C1 protein will transport it to the mitochondria where there will be a synthesis of neurosteroids (e.g., allopregnanolone and dehydroepiandrosterone) via pregnenolone. The NMDA/GABAA and nuclear receptors are acted upon by neurosteroids to promote neurogenesis (the process by which nervous system cells are formed from neural stem cells) and modulate neurotransmission (Sayeed et al., 2006). Some indices of atherogenic dyslipidemia like high TG/HDL cholesterol were reported to be positively associated with the prevalence of silent brain infarct in a neurologically-healthy population (Nam et al., 2019), indicating harmful small/dense LDL that could contribute to cerebrovascular diseases (Bittner et al., 2009). Based on the convincing links between the lipid metabolism and brain (dys)functions, we estimated the profile of lipoproteins and ratios in the brain homogenate of rats administered with CS and/or M. We observed that the CS and M neither affected the lipoprotein cholesterols nor their calculated ratios in the rat brain either when administered separately or when co-administered.

Oxidative (and nitrosative) stress is a common feature of acute or chronic neurodegenerative diseases like Alzheimer’s disease, multiple sclerosis, Parkinson’s disease, and amyotrophic lateral sclerosis (Pacher et al., 2007). Oxidative stress also provides a key link between environmental factors (e.g., heavy metals, pesticides, and herbicides) with genetic risk and endogenous factors in the pathogenic mechanisms of neurodegeneration. Disruption of the blood-brain barrier’s integrity and reactive changes in the glial elements in the CNS, which facilitates the penetration of inflammatory cells and various toxins to the site of brain injury and leads to irreversible degeneration, are caused by oxidative or nitrosative stress (Pacher et al., 2007). Moreover, various forms of acute (e.g., stroke, traumatic brain injury, and epilepsy) or chronic (e.g., Alzheimer’s disease, multiple sclerosis, Huntington’s disease, Parkinson’s disease, HIV-associated dementia, etc.) neurodegenerative disorders are caused by dysregulation of the endocannabinoid system (Bisogno & Di Marzo, 2010), as reflected by the increase or decrease in endocannabinoid content or altered CBRs expression in diseased animal or human tissues. Interestingly, the neuroprotective potentials of plant-derived cannabinoids in the CNS have been established (Pope et al., 2010), and the neuroprotection is mediated via their antioxidant property, among others (Pacher & Haskó, 2008).

In the present study, we observed that the CS increased G6PD, GPx, and SOD, but decreased NO and had no effect on MDA, CAT, GR, and AChE. Our data support the contention that CS elicits an anti-oxidative effect on the brain tissue. To understand the effect of cannabinoid-melatonergic systems interaction on the redox status of the brain, we estimated some redox parameters in the brain of rats treated with CS + M. We observed that M increased G6PD but reduced GPx, SOD, and NO in the brain of CS-treated rats. In this context, M augmented the increase in G6PD and decrease in NO but abolished the increase in GPx and SOD elicited by CS in the rat brain. This suggests that the redox effect of M in the brain of CS-treated rats is substrate-specific, which could either lead to the pro- or anti-oxidant condition. We also speculate that the increase in the G6PD (which is a second line anti-oxidant) in the brain of rats that received CS + M was a reactive response to the depletion in the first-line anti-oxidants (SOD and GPx). However, the exacerbation of CS-induced reduction of NO by M despite the pro-oxidant effect of their combination cannot be convincingly explained in this study and needs further attention.

The SOD, a ubiquitous metal-containing enzyme, converts superoxide anion into O_2_ and H_2_O_2_ (Çimen, 2008). The GPx is an enzyme family with peroxidase that protects organisms from oxidative damage by reducing lipid hydroperoxides to their corresponding alcohols and free hydrogen peroxide to water (Muthukumar et al., 2011). Reduced glutathione (GSH), which is a tripeptide molecule that consists of l-glutamate, l-cysteine, and l-glycine, is the most important antioxidant and free radical scavenger in the brain. In the presence of GPx, the GSH is oxidized (via removal of hydrogen) by hydrogen peroxide to form oxidized glutathione disulfide (GSSG), which can also be converted back to GSH by glutathione reductase (Bhabak & Mugesh, 2010). Therefore, the ratio of reduced (GSH)/oxidized (GSSH) determines the redox state of cells (Wu et al., 2004). Our observation of an increase in the brain SOD and GPx in this study and the previously reported increase in the brain GSH (Abdel-Salam et al., 2018) in rats suggest an anti-oxidative potential of both the ethanol and chloroform extracts of CS respectively.

Pathological changes in the brain cause a rapid alteration in the morphology and phagocytes behavior of microglial cells, leading to an increase in their cytotoxic responses characterized by secretion of NO, proteases, and cytokines such as tumor necrosis factor-alpha (TNF-α) and IL-1β. An increase in neuronal NO has been implicated in the endoplasmic reticulum stress and peroxynitrite-mediated oxidative/nitrosative damage (Zhu et al., 2017). Our observation of a decrease in NO without a change in MDA contrasts the previously reported increase in the MDA but unchanged NO in the brain of pentylenetetrazole-treated rats (Abdel-Salam et al., 2018). While their study suggested that CS causes lipid peroxidation even when GSH increases, our present study suggests that CS reduces nitrosative stress by reducing NO. The reduction in NO and the corresponding increase in antioxidant enzymes (e.g., G6PD, GPx, and SOD) by CS observed in our study might be responsible for the absence of lipid peroxidation (evident from the unchanged level of MDA) in CS-treated rats. These corroborate the contention that CS is neuroprotective especially by enhancing antioxidants and suppressing oxidative and nitrosative stresses.

## Conclusions

This study showed that CS and M do not alter brain lipid either when administered separately or when co-administered. Moreover, CS increases the anti-oxidant system while CS + M elevates the oxidative mechanism in the rat brain. This study has several limitations. First, we were not able to know the contributions of the CBRs and melatonin receptors (MTs) in the effect of CS-M interaction on antioxidant status. Second, we did not use different doses of M to determine whether its effect is dose-dependent in the CS-treated rat brain. However, the study’s clinical significance lies in the observation that the interaction of the cannabinoid and melatonergic systems could alter redox system. A similar study should be conducted in human so as to further our understating and advance the possible exploration of the cannabinoid-melatonergic systems in the brain.

## Supplementary Information

**Additional file 1.**

## Data Availability

The datasets used and/or analyzed during the current study are available from the corresponding author on reasonable request.

## Abbreviations

AANAT: Arylalkylamine N-acetyltransferase
AC: Atherogenic coefficient
AChE: Acetylcholinesterase
AIP: Atherogenic index of plasma
ALS: Amyotrophic lateral sclerosis
ANOVA: Analysis of variance
AOAC: Association of Official Analytical Chemists
CAT: Catalase
CB: Cannabinoid
CBR: Cannabinoid receptor
CNS: Central nervous system
CRR: Cardiovascular risk ratio
CS: *Cannabis sativa*
DNA: Deoxyribonucleic acid
G6PD: Glucose-6-phosphate dehydrogenase
GC-MS: Gas chromatography–mass spectrometry
GPx: Glutathione peroxidase
GR: Glutathione reductase
GSH: Reduced glutathione
GSSG: Glutathione disulfide
HD: Huntington’s disease
HDL-C: High-density lipoprotein-cholesterol
HFD: High-fat diet
IL: Interleukin
LDL-C: Low-density lipoprotein-cholesterol
LSD: Least significant difference
M: Melatonin
MDA: Malondialdehyde
MT: Melatonin receptor
NDLEA: National Drug Law Enforcement Agency
NO: Nitric oxide
PUFA: Polyunsaturated fatty acid
ROS: Reactive oxygen species
SEM: Standard error of mean
SFA: Saturated fatty acid
SOD: Superoxide dismutase
SPSS: Statistical Package for Social Sciences
TBARS: Thiobarbituric acid reactive substances
TC: Total cholesterol
TG: Triglyceride
THC: Tetrahydrocannabinol
TNF-α: Tumor necrosis factor-alpha
USFA: Unsaturated fatty acid
VLDL-C: Very low-density lipoprotein-cholesterol
