# Supplementary material for: Cannabis sativa and/or melatonin do not alter brain lipid but alter oxidative mechanisms in female rats
Source: J Cannabis Res. 2021 Aug 19;3:38. doi: 10.1186/s42238-021-00095-9 (PMC8377844; doi:10.1186/s42238-021-00095-9)
Supplement: Supplementary file 1 — Additional file 1. [file 42238_2021_95_MOESM1_ESM.docx]

**Table 2. Proximate analysis of CS sample used in this study**

|  | **Substance** | **Abundance (% by weight)^*^** | **Estimated energy content** (kcal/kg) |
| --- | --- | --- | --- |
| **Nutrients** | **Fiber** | 35.94 | **-** |
|  | **Carbohydrate** | 17.47 | **69.88** |
|  | **Protein** | 17.12 | **68.48** |
|  | **Total ash** | 12.51 | **-** |
|  | **Moisture** | 9.01 | **-** |
|  | **Lipids** | 7.96 | **71.64** |
|  |  |  |  |
|  | **Element** | **Amount detected (**mg/100g extract**)^#^** |  |
| **Mineral** | **Potassium** | 1506.5 |  |
|  | **Magnesium** | 202.5 |  |
|  | **Sodium** | 36.5 |  |
|  | **Iron** | 6.6 |  |
|  | **Zinc** | 3.5 |  |
|  | **Calcium** | 3 |  |

*, Average of two replicates; #, Average of three replicates; Abundance means % of the nutrients by weight; CS, *Cannabis sativa*;

The standard method of Association of Official Analytical Chemists (AOAC) was used to determine the nutrients in the CS.

**Table 3. Phytochemicals in the CS**

| **Phytochemicals** | ***Qualitative*** | ***Quantitative (mg/100g)*** |
| --- | --- | --- |
| Terpenoids | + | 14.62 |
| Phenols | + | 6.34 |
| Tannins | + | 5.32 |
| Alkaloids | + | 5.10 |
| Flavonoids | + | 4.38 |
| Saponins | + | 1.37 |
| Steroids | + | 0.48 |

CS, *Cannabis sativa*; +, detected.

**Table 4: Composition of CS determined by GC-MS**

| **S/N** | **Molecular Formula** | **Systemic Name** | **Compounds’ Trivial Name** | **Retention Time (min)** | **Relative Abundance (%)** | **Molecular Weight** |
| --- | --- | --- | --- | --- | --- | --- |
| **1** | C_19_H_36_O_2_ | 9-Octadecenoic acid, methyl ester | Elaidic acid methyl ester | 18.836 | 15.82 | 296 |
| **2** | C_19_H_34_O_2_ | 9,12-Octadecadienoic acid, methyl ester | Linoleic acid methyl ester | 18.763 | 9.01 | 294 |
| **3** | C_21_H_26_O_2_ | 6,6,9-trimethyl-3-pentylbenzo[c]chromen-1-ol | Cannabinol | 23.567 | 8.86 | 310 |
| **4** | C_17_H_34_O_2_ | Hexadecanoic acid, methyl ester | Palmitic acid methyl ester | 17.358 | 7.01 | 270 |
| **5** | C_21_H_30_O_2_ | 6,6,9-trimethyl-3-pentyl-6*a*,7,8,10*a*-tetrahydrobenzo[c]chromen-1-ol | Dela-9-tetrahydrocannabinol | 23.014 | 5.51 | 314 |
| **6** | C_16_H_28_O_2_S_2_ | 2-[1-(2-[1,3]Dithian-2-yl-ethyl)-pent-4-enyloxy]-tetrahydropyran |  | 20.889 | 4.71 | 316 |
| **7** | C_19_H_38_O_2_ | methyl octadecanoate | Methyl stearate | 19.059 | 3.78 | 298 |
| **8** | C_15_H_26_O | 1H-Cycloprop[e]azulen-4-ol,decahydro-1,1,4,7-tetramethyl- | Himbaccol | 15.869 | 3.37 | 222 |
| **9** | C_21_H_28_O_3_ | 4-(4,4-Dimethyl-7-pentyl-3,4-dihydrofuro[4,3,2-de]chromen-3-yl)-2-butanone | Cannabicoumaronone | 22.418 | 3.01 | 328 |
| **10** | C_18_H_33_ClO | 9-Octadecenoyl chloride | Oleoyl chloride | 24.253 | 2.56 | 300 |
| **`11** | C_24_H_48_O_2_ | Tetracosanoic acid | Lignoceric acid | 24.414 | 2.24 | 368 |
| **12** | C_28_H_46_O | Ergosta-7,22-dien-3-ol, (3.beta.,22E)- | 5,6-Dihydroergosterol | 24.052 | 2.23 | 398 |
| **13** | C_21_H_30_O_2_ | 2-methyl-2-(4-methylpent-3-enyl)-7-pentylchromen-5-ol | Cannabichromene | 21.448 | 2.17 | 314 |
| **14** | C_19_H_22_O_2_ | 6H-Dibenzo[b,d]pyran-1-ol, 6,6,9-trimethyl-3-propyl- | Cannabivarin | 21.775 | 2.00 | 282 |
| **15** | C_20_H_22_O_3_ | (*E*)-3-(4-butoxyphenyl)-1-(4-methoxyphenyl)prop-2-en-1-one | trans-4-Butoxy-4'-methoxychalcone | 23.815 | 1.98 | 310 |
| **16** | C_28_H_46_O | Ergosta-5,24(28)-dien-3β-ol- | Chalinasterol | 24.189 | 1.91 | 398 |
| **17** | C_26_H_33_ClO_4_ | Isophthalic acid, 4-chlorophenyl dodecyl ester | chlorotonil B | 22.282 | 1.81 | 444 |
| **18** | C_15_H_24_O | 4,12,12-trimethyl-9-methylidene-5-oxatricyclo[8.2.0.0^4,6^]dodecan | Caryophyllene oxide | 14.291 | 1.73 | 220 |
| **19** | C_20_H_40_O | 3,7,11,15-tetramethylhexadec-2-en-1-ol | Phytol | 18.927 | 1.55 | 296 |
| **20** | C_11_H_18_O_2_ | 2,6,8-Trimethylbicyclo[4.2.0]oct-2-ene-1,8-diol |  | 16.229 | 1.54 | 182 |
| **21** | C_25_H_36_O_4_ | 2-*O*-(2-methylpropyl) 1-*O*-tridec-2-ynyl benzene-1,2-dicarboxylate | Phthalic acid | 16.699 | 1.19 | 400 |
| **22** | C_15_H_18_O_3_ | 4-hydroxy-6-methoxyspiro[1,2-dihydroindene-3,4'-cyclohexane]-1'-one | Cannabispiran | 21.290 | 1.00 | 246 |
| **23** | C_15_H_28_O | Cyclopentadecanone | Normuscone | 16.461 | 0.98 | 224 |
| **24** | C_11_H_16_ | 3-ethenyl-7,7-dimethylbicyclo[4.1.0]hept-3-ene |  | 19.329 | 0.95 | 148 |
| **25** | C_21_H_42_O_2_ | Methyl 18-methylnonadecanoate | Nonadecanoic acid | 21.069 | 0.94 | 326 |
| **26** | C_15_H_26_O | (7a-Isopropenyl-4,5-dimethyloctahydroinden-4-yl)methanol |  | 13.354 | 0.92 | 222 |
| **27** | C_30_H_46_O_9_ | -dodecahydro-1*H*-cyclopenta[a]phenanthren-17-yl]-2*H*-furan-5-one | Emicymarin | 18.047 | 0.91 | 550 |
| **28** | C_15_H_24_O | 4,4-dimethyltetracyclo[6.3.2.0^1,8^.0^2,5^]tridecan-9-ol - |  | 14.826 | 0.87 | 220 |
| **29** | C_19_H_36_O_2_ | Cyclopentanetridecanoic acid, methyl ester | Methyl dihydrochaulmoograte | 15.390 | 0.85 | 296 |
| **30** | C_27_H_44_O_3_ | 1alpha,25-dihydroxyvitamin D_3_ | Calcitriol | 21.368 | 0.80 | 416 |
| **31** | C_15_H_24_O | 1,5,5,8-tetramethyl-12-oxabicyclo[9.1.0]dodeca-3,7-diene |  | 14.566 | 0.70 | 220 |
| **32** | C_19_H_26_O_2_ | -6,6,9-trimethyl-3-propyl-6*a*,7,8,10*a*-tetrahydrobenzo[c]chromen-1-ol | Delta-9-tetrahydrocannabivarin | 21.191 | 0.67 | 286 |
| 33 | C_22_H_34_O_3_ | **18-Oxokauran-17-yl acetate** |  | 16.602 | 0.67 | 346 |
| **34** | C_14_H_22_O | 4-(6,6-Dimethyl-2-methylenecyclohex-3-enylidene)pentan-2-ol |  | 14.905 | 0.61 | 206 |
| **35** | C_17_H_32_O_2_ | 7-Hexadecenoic acid, methyl ester |  | 16.999 | 0.60 | 254 |
| **36** | C_3_H_4_O_4_ | Propanedioic acid, | Malonic acid | 19.203 | 0.58 | 104 |
| **37** | C_15_H_26_O | "1-Isopropyl-4,7-dimethyl-1,3,4,5,6,8a-hexahydro-4a(2H)-naphthalenol | Cubenol | 16.882 | 0.52 | 222 |
| **38** | C_12_H_22_O | Cyclohexane, 1,1'-oxy bis-cyclohexane | Dicyclohexyl ether | 10.316 | 0.51 | 182 |
| **39** | C_16_H_22_OS | 2,6,6-Trimethyl-3-(phenylthio) cyclohept-4-enol |  | 16.807 | 0.49 | 262 |
| **40** | C_21_H_44_OSi | 1-Ethyl-1-tetradecyloxy-1-silacyclohexane |  | 24.922 | 0.48 | 340 |
| **41** | C_19_H_30_O_2_ | -10,13-dimethyl-2,3,4,7,8,9,11,12,14,15,16,17-dodecahydro-1*H*-cyclopenta[a]phenanthrene-3,17-diol | 3beta-hydroxy-5-androstene-17-ol | 19.498 | 0.42 | 290 |
| **42** | C_15_H_24_O | cis-Z-.alpha.-Bisabolene epoxide | Oxirane | 14.791 | 0.41 | 220 |
| **43** | C_20_H_40_O | 3,7,11,15-Tetramethyl-2-hexadecen-1-ol | Phytol | 16.393 | 0.38 | 296 |
| **44** | C_15_H_24_O | [(2E)-2-(4,7-Dimethyl-,4,4a,5,6,8a-hexahydro-1(2H)-naphthalenylidene)-1-propanol] | Lanceol, cis; | 14.427 | 0.37 | 220 |
| **45** | C_11_H_12_O_2_ | 3,3,7-trimethyl-2-benzofuran-1-one |  | 13.052 | 0.35 | 176 |
| **46** | C_19_H_34_O_2_ | methyl octadeca-7,10-dienoate |  | 19.718 | 0.33 | 294 |
| **47** | C_15_H_26_O | 1,1,4,7-tetramethyl-2,3,4*a*,5,6,7,7*a*,7*b*-octahydro-1*aH*-cyclopropa[e]azulen-4-ol | Globulol | 15.033 | 0.32 | 222 |
| **48** | C_15_H_24_ | -4,11,11-trimethyl-8-methylidenebicyclo[7.2.0]undec-4-ene | Caryophyllene | 12.522 | 0.25 | 204 |
| **49** | C_21_H_30_O_3_ | 17-acetyl-16-hydroxy-10,13-dimethyl-1,2,6,7,8,9,11,12,14,15,16,17-dodecahydrocyclopenta[a]phenanthren-3-one | Pregn-4-ene-3,20-dione, 16-hydroxy-, (16.alpha) | 24.586 | 0.21 | 330 |
| **50** | C_10_H_14_O | Bicyclo[3.1.1]hept-2-en-6-one, 2,7,7-trimethyl | Chrysanthenone | 11.612 | 0.18 | 150 |
| **51** | C_15_H_24_ | 5,8*a*-dimethyl-3-propan-2-ylidene-1,2,4,4*a*,7,8-hexahydronaphthalene | Naphthalene | 13.282 | 0.14 | 204 |
| **52** | C_27_H_38_O_9;_ | 4-pregnen-21-ol-3, 20-dione glucosiduronate | 21-hydroxy-4-pregnene-3,20-dione 21-glucoside | 25.417 | 0.11 | 492 |
| **53** | C_25_H_42_O_2_ | Cyclopropanebutanoic acid |  | 25.196 | 0.09 | 374 |

CS, *Cannabis sativa*; GC-MS, gas chromatography – mass spectrometry. The procedure for the CG-MS was as previously described ^63^
